# A Fungal Secretome Adapted for Stress Enabled a Radical Wood Decay Mechanism

**DOI:** 10.1128/mBio.02040-21

**Published:** 2021-08-17

**Authors:** Jesus Castaño, Jiwei Zhang, Mowei Zhou, Chia-Feng Tsai, Joon Yong Lee, Carrie Nicora, Jonathan Schilling

**Affiliations:** a Bioproducts and Biosystems Engineering, University of Minnesota, Saint Paul, Minnesota, USA; b Environmental Molecular Sciences Laboratory, Pacific Northwest National Laboratory, Richland, Washington, USA; c Biological Sciences Division, Pacific Northwest National Laboratory, Richland, Washington, USA; d Plant and Microbial Biology, University of Minnesota, Saint Paul, Minnesota, USA; e Marine and Coastal Research Institute, Invemar, Santa Marta, Colombia; Cornell University

**Keywords:** brown rot fungi, ROS tolerance, glycosyl hydrolases, proteomics

## Abstract

Brown rot fungi release massive amounts of carbon from forest deadwood, particularly at high latitudes. These fungi degrade wood by generating small reactive oxygen species (ROS) to loosen lignocellulose, to then selectively remove carbohydrates. The ROS mechanism has long been considered the key adaptation defining brown rot wood decomposition, but recently, we found preliminary evidence that fungal glycoside hydrolases (GHs) implicated in early cell wall loosening might have been adapted to tolerate ROS stress and to synergize with ROS to loosen woody lignocellulose. In the current study, we found more specifically that side chain hemicellulases that help in the early deconstruction of the lignocellulosic complex are significantly more tolerant of ROS in the brown rot fungus Rhodonia placenta than in a white rot fungus (Trametes versicolor) and a soft rot fungus (Trichoderma reesei). Using proteomics to understand the extent of tolerance, we found that significant oxidation of secreted R. placenta proteins exposed to ROS was less than half of the oxidation observed for T. versicolor or T. reesei. The principal oxidative modifications observed in all cases were monooxidation and dioxidation/trioxidation (mainly in methionine and tryptophan residues), some of which were critical for enzyme activity. At the peptide level, we found that GHs in *R. placenta* were the least ROS affected among our tested fungi. These results confirm and describe underlying mechanisms of tolerance in early-secreted brown rot fungal hemicellulases. These enzymatic adaptations may have been as important as nonenzymatic ROS pathway adaptations in brown rot fungal evolution.

## INTRODUCTION

Brown rot fungi access and metabolize wood structural carbohydrates (cellulose and hemicellulose) without removing significant amounts of lignin (1, 2). Relative to white rot fungi that remove lignin at rates similar to or higher than those for carbohydrates, carbohydrate-selective brown rot pathways may provide efficient means for industrial biomass deconstruction. Brown rot fungi also presumably shunt more wood C to soil as lignin residues, with enormous implications for C sequestration in forests (3), particularly in higher-latitude forests where brown rot is prevalent (4).

The mechanism used by brown rot fungi relies on reactive oxygen species (ROS), specifically hydroxyl radicals, that are generated by small diffusible agents producing the Fenton reaction (Fe^2+^ + H_2_O_2_ → Fe^3+^ + HO^−^ + HO·) (5). This intense, oxidative first step is followed by a delayed second wave of carbohydrate-active enzymes (CAZys) (6–8), presumably to loosen lignocellulose oxidatively and minimize the oxidative denaturation of hydrolytic enzymes (8, 9). This two-step approach is a logical “damage control” explanation for the brown rot mechanism, but some CAZys, especially glycosyl hydrolases (GHs) associated with side chain hemicellulose degradation (arabinan and galactan), do not follow this delayed pattern (8). These early GHs have been implicated alongside ROS as playing a critical role in loosening wood cell walls (10) and may be as important in the story of brown rot evolution as the adaptation of ROS.

We hypothesized that early-decay GHs secreted by brown rot fungi would be adapted to tolerate ROS stress, relative to the GHs of other wood-degrading fungi, specifically their close ancestors, white rot fungi. Two lines of evidence support our hypothesis. (i) Rhodonia placenta’s GHs are 2.5 times more glycosylated than those in non-brown rot fungi (11), a posttranslational modification (PTM) that may help shield enzymes from oxidative damage. (i) We recently showed that GHs in R. placenta were more tolerant of ROS than the same enzymes in the industrial “workhorse” Trichoderma reesei (12). As a distant soft rot fungus that does not effectively degrade wood in nature, T. reesei produces high levels of cellulases and hemicellulases in a rather nonoxidative environment (13, 14). The oxidative tolerance of brown rot GHs has not yet been tested in comparison with white rot fungi, and the protein structural mechanisms of oxidative tolerance have not been defined.

There are several key reasons why this comparison with a white rot fungus was critical for our study. (i) White rot fungi also use ROS during wood decomposition, although ROS-linked genes tend to be expressed at late decay stages (15). (ii) White rot fungi are closely related to brown rot fungi and average nearly 3 times more GH genes in their genomes (16–18). (iii) Despite lower GH gene counts than white rot fungi, brown rot fungi have been shown to express GHs at far higher levels (15), and they have genes that code for proteins involved in oxidative stress responses. These observations, collectively, could provide evidence supporting an alternative hypothesis: GHs, aided by stress response proteins, could be overexpressed/secreted by brown rot fungi into an oxidatively stressful environment for the sake of low but energetically acceptable sugar yields.

To test the hypothesis that brown rot fungi have adapted ROS-tolerant GHs relative to white rot fungi, we subjected crude protein extracts from the brown rot fungus *Rhodonia placenta* and the white rot fungus Trametes versicolor to oxidative treatment using the Fenton reaction (Fe^2+^ + H_2_O_2_ → Fe^3+^ + HO^−^ + HO·), including *T. reesei*, to scale similarities and tie to previous work (12). Next, we evaluated the residual activity of the key side chain hemicellulases α-l-arabinofuranosidase and α-d-galactosidase, using these activities to frame deeper analyses to define mechanisms of tolerance. In these mechanistic analyses, we analyzed the effects of the same ROS oxidative treatment within the secretomes of all three fungi, focusing on degradation and modification events such as kynurenine formation, monooxidation, and carbonylation. These tests clearly supported our hypothesis that *R. placenta* adapted these key enzymes to endure ROS stress, relative to white rot and soft rot fungi, and that the principal oxidative modifications were monooxidation and dioxidation/trioxidation in methionine and tryptophan residues. Next, using protein modeling and molecular docking analyses, we showed how oxidation could inactivate the α-l-arabinofuranosidases and α-d-galactosidases in *T. reesei* and T. versicolor. With this, we present high-resolution insight into how ROS affect the GH proteins of these fungi.

## RESULTS

### Activity assays confirmed ROS tolerance in brown rot enzymes.

**FIG 1:**
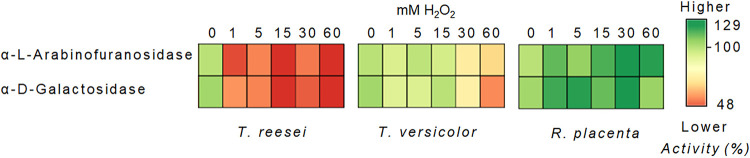
Residual activity after various degrees of ROS exposure in *T. reesei*, *T. versicolor*, and *R. placenta*, shown as a heat map. The concentration of FeSO_4_ was constant (1 mM) as H_2_O_2_ was added, and no FeSO_4_ was added to the control. The activity value at 0 mM H_2_O_2_ equals 100%.

Side chain hemicellulase activities in *R. placenta* (brown rot fungus) were, in line with our hypothesis, more tolerant of ROS than those in Trichoderma reesei (soft rot) and a more closely related white rot fungus, *Trametes versicolor* (Fig. 1; see also Fig. S1A in the supplemental material). Principal-component analysis (PCA) showed that this difference was significant (Fig. S1B). The residual activity levels in α-l-arabinofuranosidase and α-d-galactosidase at high concentrations of H_2_O_2_ were reduced for *T. versicolor* up to 40% and for *T. reesei* up to 50%. Possible intrinsic metabolite interference was ruled out by buffer exchange. The tolerance pattern also held when the bulk extracts were deglycosylated (Fig. S1C), indicating that excessive glycosylation in *R. placenta* GHs (>2.5 times the typical cellulolytic enzyme levels) (11) seems not to be the main structural feature enabling ROS tolerance. However, it is important to note that bulk extract experiments hinder the possibility of verifying that full deglycosylation has been carried out, and as a result, to rule out any protective role of glycosylation in the tolerance of ROS, assays with individually purified proteins are needed. In addition, we also evaluated the polygalacturonase activity changes after oxidation in all three fungi since we had obtained differential behaviors previously between *R. placenta* and *T. reesei* (12). However, the results in this study did not show important differences between *R. placenta* and *T. versicolor* (Fig. S2).

10.1128/mBio.02040-21.2 FIG S1 (A) Enzyme oxidative tolerance as relative GH activities. Damage is due to treatment with the Fenton reagents. 0_A_, control without Fe^2+^ or H_2_O_2_; 0_B_, control with only 1 mM Fe^2+^ and catalase added from the beginning of the experiment; 0_C_, control with only 1 mM FeSO_4_ and catalase (2,990 U) added after 1 h of incubation. Values of 1, 5, 15, 30, and 60 mM refer to the concentrations of H_2_O_2_ used in each treatment, in which the FeSO_4_ concentration was fixed at 1 mM. The values are shown as activities of treated samples relative to that of the untreated control (0_A_) (means ± standard deviations; *n *= 3). Bars with the same lowercase letters in the same series are not significantly different (*P > *0.05). Initial specific enzyme activities are embedded as units per milligram within the controls. (B) Principal-component analysis of the enzyme activity reduction after oxidative treatment shows how the effect is highly influenced by the type of fungus. (C) Enzyme oxidative tolerance in glycosylated and deglycosylated bulk extracts of *T. reesei*, *T. versicolor*, and *R. placenta*. ROS were generated through the Fenton reaction (30 mM H_2_O_2_ plus 1 mM Fe^2+^). The values represent the average residual activities (means ± standard deviations; *n *= 3) with respect to a control in which neither Fe^2+^ nor H_2_O_2_ was added. The stars indicate significant differences (*P* > 0.05) between deglycosylated and glycosylated samples. Download FIG S1, TIF file, 0.2 MB. Copyright © 2021 Castaño et al. 2021 Castaño et al. https://creativecommons.org/licenses/by/4.0/ This content is distributed under the terms of the Creative Commons Attribution 4.0 International license.

10.1128/mBio.02040-21.3 FIG S2 Residual pectinase activity analysis after performing oxidative treatment with the Fenton reaction (Fe^2+^ + H_2_O_2_ → Fe^3+^ + HO^−^ + HO·). The loss of activity in *T. reesei* could not be associated with specific amino acid oxidation events through proteomics experiments. Download FIG S2, TIF file, 0.9 MB. Copyright © 2021 Castaño et al. 2021 Castaño et al. https://creativecommons.org/licenses/by/4.0/ This content is distributed under the terms of the Creative Commons Attribution 4.0 International license.

### Proteomics showed no clear links between tolerance and protein abundances.

This clear differential effect on enzyme activities supported deeper proteomics to define the mechanisms of tolerance. Specifically, in addition to glycosylation tested in activity assays, we compared protein degradation (as concentration losses) as well as more resolved quantifications of specific amino acid oxidation.

**FIG 2:**
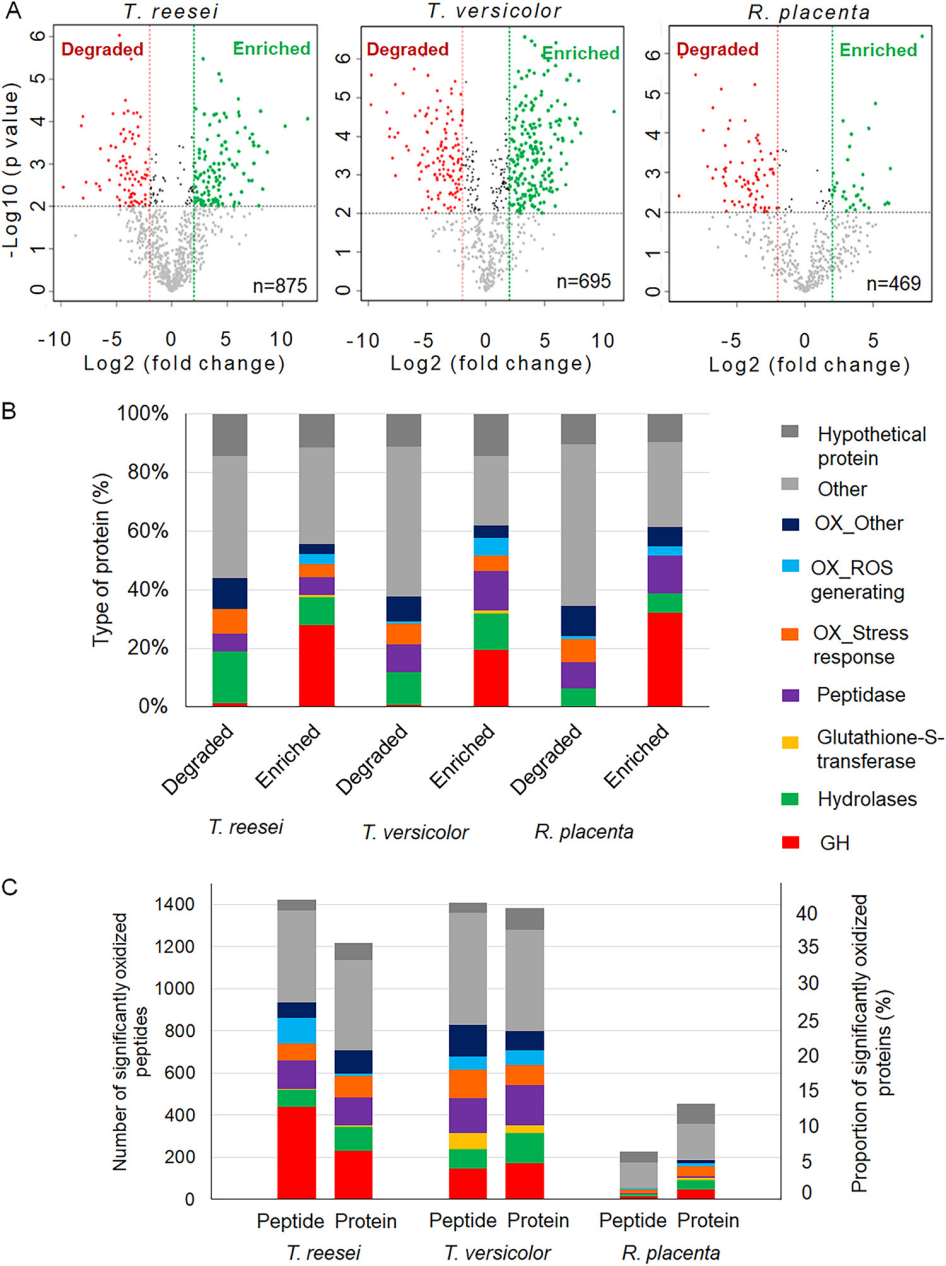
Degradation within fungal secretomes. (A) Concentration changes observed as a result of oxidative treatment in all three fungi (log_2_ fold change [FC] of >2.0; *P* value of <0.01). The degradation rates were 9.6%, 18.3%, and 16.8% for *T. reesei*, *T. versicolor*, and *R. placenta*, respectively. (B) Types of proteins degraded (lower concentration after treatment) or enriched (higher concentration after treatment) after oxidative treatment. A detailed list of proteins that significantly changed concentrations after oxidative treatment can be found in Data Set S1 in the supplemental material. (C) Proportion of significantly oxidized peptides and proteins (*P* value of <0.01) by type of enzyme (at least one peptide is bearing an oxidative modification for each protein). The sum of the percentages for each fungus gives the percentage of total proteins oxidized in each case (log_2_ FC of >2.0). A detailed list of significantly oxidized proteins and peptides can be found in Data Set S2 in the supplemental material.

We found no clear correlation of ROS-induced activity losses with overall protein degradation, including stress response proteins. The smallest amount of protein degradation was recorded for *T. reesei* (Fig. 2A), the fungus with the greatest ROS-induced loss of residual side chain hemicellulase activity (Fig. 1A). The GH proteins in all three fungi were enriched (not degraded, thus increasing the concentration) to over 20% of the secretome, with only 1% degradation in *T. reesei* and *T. versicolor* (Fig. 2B). The only side chain hemicellulase degraded in any tested fungus was a GH27 α-d-galactosidase in *T. reesei* (jgi|Trire|55999; 256-fold difference). Stress response oxidoreductases in *R. placenta*, such as manganese superoxidase, glutathione peroxidase, fumarate reductase, and peroxiredoxin, were significantly degraded by ROS and not enriched as would be expected if they enabled tolerance (Fig. 2B). Similar proteins in *T. versicolor* and *T. reesei* had more mixed degradation/enrichment patterns, including a slight enrichment of a group with a similar function, glutathione *S*-transferases.

10.1128/mBio.02040-21.9 DATA SET S1 Protein concentration changes after the oxidative treatment measured by proteomics. Download Data Set S1, XLSX file, 0.7 MB. Copyright © 2021 Castaño et al. 2021 Castaño et al. https://creativecommons.org/licenses/by/4.0/ This content is distributed under the terms of the Creative Commons Attribution 4.0 International license.

10.1128/mBio.02040-21.10 DATA SET S2 Oxidative changes at the protein and peptide level identified by proteomics. Download Data Set S2, XLSX file, 0.5 MB. Copyright © 2021 Castaño et al. 2021 Castaño et al. https://creativecommons.org/licenses/by/4.0/ This content is distributed under the terms of the Creative Commons Attribution 4.0 International license.

### Proteomics showed a clear link between tolerance and oxidation events.

Having ruled out glycosylation, degradation susceptibility, and stress response proteins, we found instead that ROS sensitivity in *R. placenta* was best explained by more resolved oxidative damage not apparent in coarse protein counts. First, we found significant (α < 0.05) increases in the numbers of oxidized proteins after oxidative treatment for *T. reesei* and *T. versicolor* (Fig. S3) but not *R. placenta*. Most of the oxidation events led to full amino acid oxidations (Fig. S4). Second, at both the peptide and the protein levels, we found more oxidation in *T. reesei* (35%) and *T. versicolor* (40%) than in *R. placenta* (13%) (Fig. 2C).

10.1128/mBio.02040-21.4 FIG S3 Oxidation dynamics I (oxidation event count). (A) Oxidized protein count. Only the absence or presence of proteins with at least one oxidized peptide was counted regardless of the extent of oxidation. (B) Oxidized peptide count. Only the absence or presence of peptides with oxidized residues was counted regardless of the extent of oxidation. Both bar graphs show means ± standard deviations (*n *= 3). The stars indicate significant differences between untreated and treated samples for the same fungus. Download FIG S3, TIF file, 0.2 MB. Copyright © 2021 Castaño et al. 2021 Castaño et al. https://creativecommons.org/licenses/by/4.0/ This content is distributed under the terms of the Creative Commons Attribution 4.0 International license.

10.1128/mBio.02040-21.5 FIG S4 Extent of oxidation using spectral counts of either proteins (A) or peptides (B). Four categories were considered: 0 to 25%, 25 to 50%, 50 to 75%, and 75 to 100%. Differences between control and treated samples within each fungus for the categories 25 to 50%, 50 to 75%, and 75 to 100% were significant in most cases (*). Differences between fungi for each category were significant only for the category 75 to 100% (ⱡ), which means that after ROS exposure, significantly higher proportions of peptides and proteins in *T. reesei* and *T. versicolor* reach full oxidation than in *R. placenta* (means ± standard deviations; *n *= 3). Download FIG S4, TIF file, 0.1 MB. Copyright © 2021 Castaño et al. 2021 Castaño et al. https://creativecommons.org/licenses/by/4.0/ This content is distributed under the terms of the Creative Commons Attribution 4.0 International license.

The specific proteins and amino acids oxidized also reveal a clear distinction in the early GHs, specifically side chain hemicellulases. As opposed to 58 (58.6%) and 35 (54.7%) GHs with oxidative damage in *T. reesei* and *T. versicolor*, respectively, *R. placenta* had only 7 (14.9%) GHs affected, none of which was a side chain hemicellulase; the percentages refer to the proportions of total GHs affected. In *T. reesei*, the 58 GHs oxidized by ROS included endoglucanases, exo-acting cellulases, β-glucosidases, xylanases, mannosidases, as well as the side chain hemicellulases α-d-galactosidase (GH27 and GH36) and α-l-arabinofuranosidase (GH54 and GH43). The higher level of oxidation in the peptide-level analyses also implied more susceptible “hot spots” for oxidation in *T. reesei* GHs (Fig. 2C). In *T. versicolor*, we found a similar pattern at the protein level, where the 35 GH proteins oxidized included endoglucanases, exo-acting cellulases, β-glucosidases, mannosidases, polygalacturonases (GH28), as well as α-d-galactosidases (GH27) and α-l-arabinofuranosidases (GH51). In *R. placenta*, only 7 GH proteins were oxidized by ROS, 5 of which were related to the metabolism of chitin and other glycans. The other 2 included a potential endoglucanase (GH5) and a pectinase (GH28).

At this point, it is important to mention that although high relative abundances of α-d-galactosidases and α-l-arabinofuranosidases in one fungus, relative to another, could influence its susceptibility to become oxidized and consequently lose its activity, no clear relationship was observed (Data Set 1). The quantitation using label-free proteomics is relative, forcing abundance ranks to be “binned” into quartiles, but this enables some capacity to rank protein abundances within a sample. By quartile, with quartile 1 (Q1) being the most abundant category relative to Q2, Q3, and Q4, we saw that the numbers of GHs (by the amount of protein and not by the type of protein) in quartiles 1 and 2 for each fungal secretome were 6.40% for *T. reesei*, 2.59% for *T. versicolor*, and 2.99% for *R. placenta* (see Data Set 1 for calculations). More specifically, within these GHs, we found that the majority of α-l-arabinofuranosidases and α-d-galactosidases for *T. reesei* were in Q1 and Q2, at higher abundances than those enzymes in either *T. versicolor* or *R. placenta* (primarily in Q3 and Q4). If higher abundances of specific proteins were to predispose them to more oxidative damage, this would have led to higher oxidative damage in *T. reesei* GHs, including α-l-arabinofuranosidases and α-d-galactosidases, and lower protein damage at similar levels in *T. versicolor* or *R. placenta*. Instead, we saw higher damage in *T. versicolor* than in *R. placenta*. This implies a structural basis for ROS tolerance in brown rot *R. placenta* GHs.

**FIG 3:**
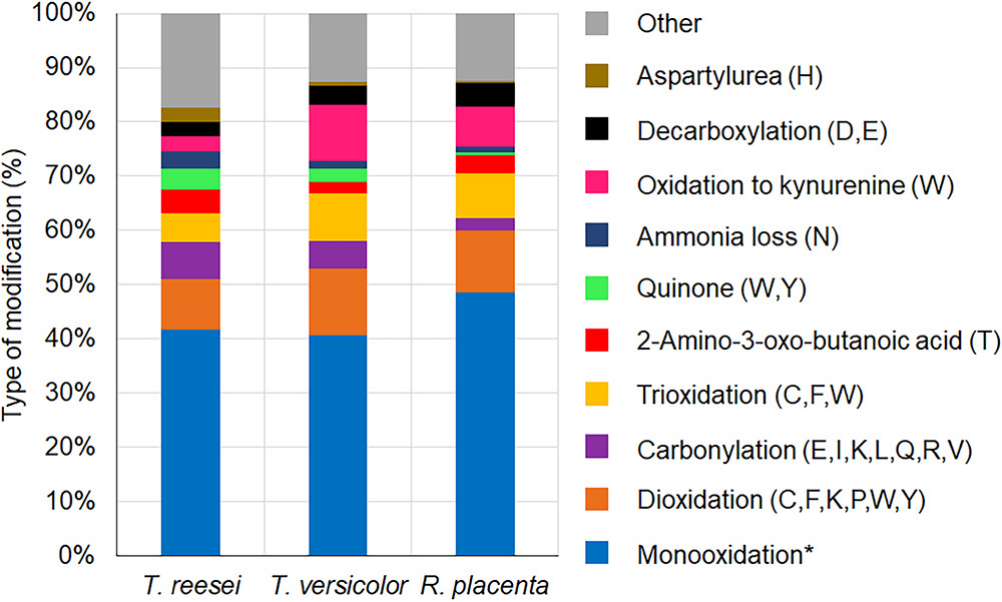
Proportions of the types of significant oxidative modifications found in *T. reesei*, *T. versicolor*, and *R. placenta* after Fenton reaction treatment (log_2_ FC of >2.0 for all peptides). *, monooxidation occurred in all 20 natural amino acids.

Going deeper, to the resolution of individual amino acids, we found that the main modifications among these three fungi were monooxidation and dioxidation (Fig. 3), which occurred most often in methionine and tryptophan amino acids (Fig. S5). The 307 significantly oxidized proteins in *T. reesei* were represented by 1,424 peptides, within which 41.7% of modifications were monooxidation events largely in methionine (25.3%), tryptophan (12.9%), and tyrosine (8.5%). Other notable modifications were dioxidation (9.3%) and carbonylation (6.8%). In *T. versicolor*, there were 280 significantly oxidized proteins represented by 1,410 peptides, within which 40.6% were monooxidations largely in tryptophan (24.8%), methionine (17.0%), and cysteine (7.1%). Finally, in *R. placenta*, there were only 66 significantly oxidized proteins represented by 226 peptides, with most modifications (again dominated by monooxidation [48.6%]) occurring in methionine (21.4%), tryptophan (19.1%), and cysteine (7.0%).

10.1128/mBio.02040-21.6 FIG S5 Distribution of significantly and uniquely oxidized residues in *T. reesei*, *T. versicolor*, and *R. placenta* (log_2_ FC of >2.0 for all peptides). Download FIG S5, TIF file, 0.4 MB. Copyright © 2021 Castaño et al. 2021 Castaño et al. https://creativecommons.org/licenses/by/4.0/ This content is distributed under the terms of the Creative Commons Attribution 4.0 International license.

### Molecular docking implicated key oxidative damage in amino acids.

**FIG 4:**
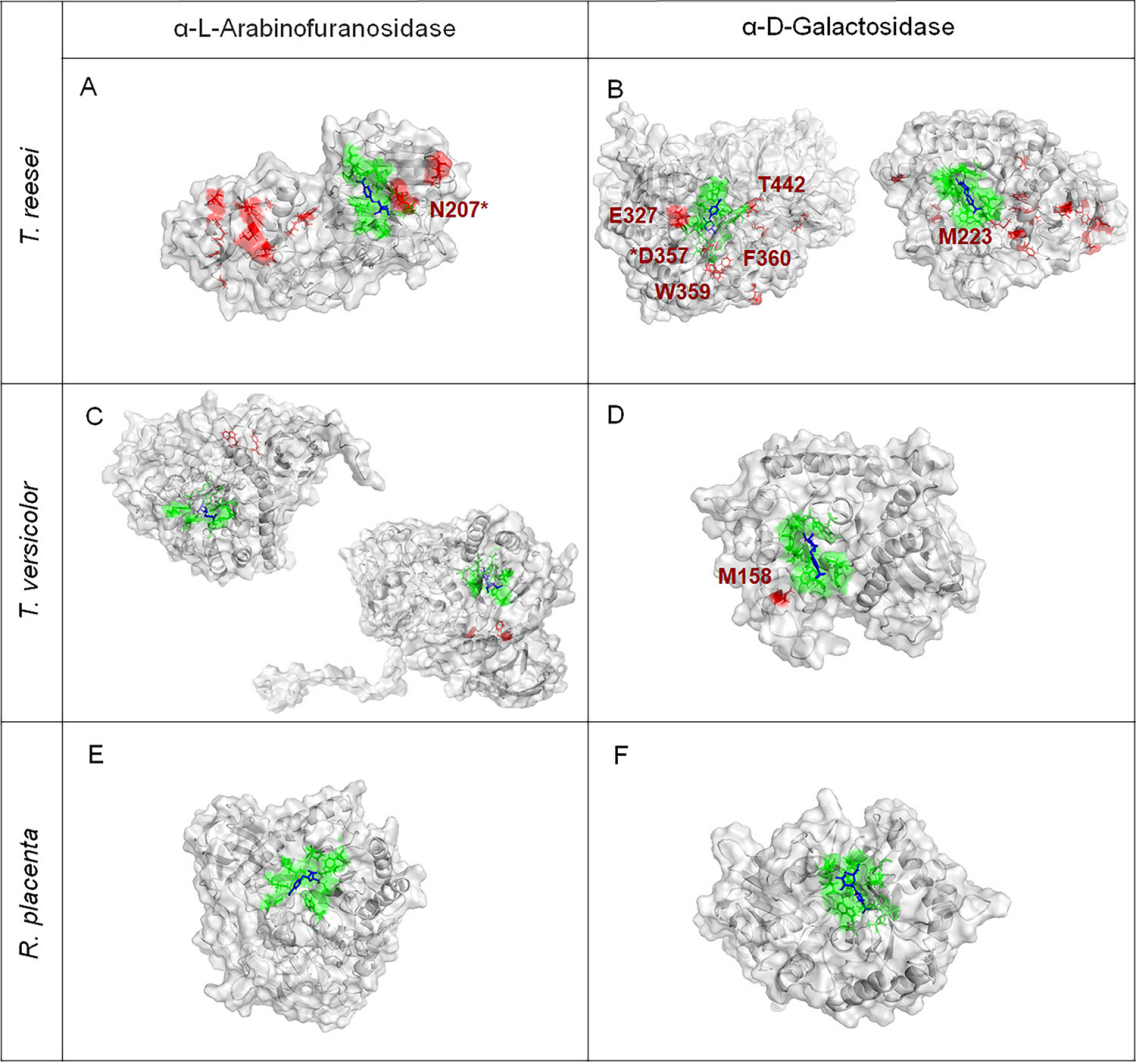
Active substrate poses for some α-l-arabinofuranosidases and α-d-galactosidases in *T. reesei*, *T. versicolor*, and *R. placenta*. The substrates (blue) interact with the binding and catalytic residues (green). The residues in red correspond to oxidized amino acids in each protein. Residues with an asterisk correspond to oxidized residues that are also predicted to be binding residues. A list of all the interacting residues found in the binding pocket can be found in Table S2 in the supplemental material. The visualizations were obtained with PyMOL. (A) α-l-Arabinofuranosidase GH54 (jgi|Trire2|55319); (B) α-d-galactosidases GH36 (jgi|Trire2|124016) (left) and GH27 (jgi|Trire2|72632) (right); (C) α-l-arabinofuranosidases GH51 (jgi|Trave1|172787) (left) and GH51 (jgi|Trave1|59914) (right); (D) α-d-galactosidase GH27 (jgi|Trire2|60477); (E) α-l-arabinofuranosidase GH51 (jgi|Pospl1|100251); (F) α-d-galactosidase GH27 (jgi|Pospl1|98662).

Finally, to correlate the loss of enzyme activity and the oxidative damage observed in this study, we identified the location and role of the oxidized residues in the enzymes α-l-arabinofuranosidase and α-d-galactosidase in *T. reesei* and *T. versicolor*. Using molecular docking, we predicted the binding poses of the substrates associated with these enzymes and identified the binding residues. As expected for most of the enzymes, many of the oxidized residues were located either within the active site or very close to it (Fig. 4). For comparison, we included GHs highly expressed in *R. placenta*. While the abundance of red zones/molecule indicating oxidation is clear for *T. reesei* and, to a lesser extent, *T. versicolor*, no oxidation was observed in *R. placenta* enzymes.

10.1128/mBio.02040-21.8 TABLE S2 Interacting residues in the catalytic pocket predicted by molecular docking analysis. Download Table S2, DOCX file, 0.01 MB. Copyright © 2021 Castaño et al. 2021 Castaño et al. https://creativecommons.org/licenses/by/4.0/ This content is distributed under the terms of the Creative Commons Attribution 4.0 International license.

## DISCUSSION

These results imply that enzyme adaptations to ROS stress, specifically oxidation tolerance in methionine and tryptophan residues, may have been just as integral in the evolution of brown rot fungi as adaptations implicated in ROS production. Our results confirm the ROS tolerance of *R. placenta* GHs expressed in early decay stages, relative to distant soft rot ancestor *T. reesei* (12), but more importantly distinguish this GH tolerance relative to that of another wood-degrading fungus, the white rot type *T. versicolor*. Relevant to what we know about general patterns of wood deconstruction in both white and brown rot fungi, this is most relevant among side chain hemicellulases. Both brown and white rot fungi hydrolyze hemicellulose enzymatically in the earliest wood decay stages (19), presumably as a structural prerequisite to gain ingress into the lignocellulose matrix. White rot fungi, however, delay the expression of most oxidative pathways relative to brown rot fungi that express and then downregulate lignocellulose oxidation (15). It makes sense that brown rot GHs involved in loosening lignocellulose, in tandem with ROS (10), would require greater ROS tolerance than white rot GHs.

We found that these unique tolerance mechanisms in GHs were not linked to presence/absence measures of glycosylation, protein degradation, and/or stress response proteins, commonly reported as enabling ROS tolerance in other organisms. Although excess glycosylation can act to shield enzymes against ROS damage (20–22), we found that glycosylation did not, at least as a direct factor, explain ROS tolerance. Protein degradation also failed to correlate. Others have shown that protein degradation can occur either as the direct result of spontaneous protein cleavage, induced by oxidative damage (23), or as the result of enhanced proteasome-mediated proteolysis, enhanced by oxidative modifications within the targets (24). In our study, however, only one side chain hemicellulase (GH27) was degraded to the point of loss after oxidative treatment, implying that the presence/absence measure of degradation was too coarse to explain activity losses. Finally, stress response proteins present in these fungal genomes, including oxidases, glutathione *S*-transferases, and thioredoxins, have been commonly implicated in ROS detoxification (25, 26). In this study, however, they represented one of the groups of enzymes most vulnerable to ROS. This suggests that ROS not only may affect the catalytic activity of GHs but also may inactivate the enzymes meant to protect against oxidative stress, exasperating ROS damage.

Instead of coarser presence/absence measures, we found, using deeper assessments of the fungal secretome, that specific oxidation events likely disrupted GH function in non-brown rot strains. In *T. reesei*, the α-l-arabinofuranosidase GH54 (jgi|Trire2|55319) binding residue N207 suffered significant ammonia loss after oxidative treatment. Although ammonia loss is not a direct oxidative modification, it is influenced partially by the structure and stability of the protein (27); thus, it can be exacerbated by structural changes induced during oxidation (28). Additionally, N207 is close to the predicted catalytic residues D204 and E206 (29), which might affect substrate positioning with respect to these residues and limit catalytic activity (other binding residues can be seen in Table S2 in the supplemental material). For the α-d-galactosidase GH36 in *T. reesei* (jgi|Trire2|124016), we also found significantly oxidized residues (E327, W359, F360, and T442) located close to the binding pocket (Fig. 4B). For instance, T442 and E327 are next to the binding residues R443 and W326, respectively (Table S2). Additionally, the active-site residue D357 was found to suffer decarboxylation after oxidative treatment. Decarboxylation is one of several modifications that proteins can undergo after oxidation (30, 31). In this case, this decarboxylated aspartic residue would lose its ability to form hydrogen bonds between its side chain and the substrate as well as salt bridges that would contribute to stabilizing K476, another binding residue (32). Finally, for the α-d-galactosidase GH27 (jgi|Trire2|72632), we found an oxidation event in M223 (Fig. 4B), immediately adjacent to the binding residue R222.

Similarly, in *T. versicolor*, we found that α-l-arabinofuranosidases and α-d-galactosidases were affected after oxidative treatment. In the α-l-arabinofuranosidase GH51 (jgi|Trave1|172787), the residues W398 and Y406 were affected by kynurenine oxidation and dioxidation, respectively (Fig. 4C). For GH51(jgi|Trave1|59914), the residues M221, F266, and M483 were affected by monooxidation (Fig. 4C). These residues were located away from the active site in both proteins, but their oxidation could still destabilize and denature a protein. For instance, tyrosine, tryptophan, and methionine oxidation has been shown to be a key factor in protein aggregation (23, 33, 34). For the α-d-galactosidase GH27 (jgi|Trave1|60477), the highly conserved residue M158 was significantly oxidized (Fig. 4D). This methionine oxidation could lead to the unraveling of one of the α-helices in the (β/α)_8_ barrel topology of the enzyme (35), which in turn might destabilize the adjacent loop containing several active-site residues. It has been found that methionine oxidation can enhance asparagine deamidation even when it is located more than 100 residues away from the oxidized methionine (28), triggering protein degradation and denaturation (36, 37) and contributing to the loss of enzyme activity.

Contrary to these oxidation weak points in *T. reesei* and *T. versicolor* side chain hemicellulases, we did not find any significant oxidative modifications in the same enzymes in *R. placenta*. We also found similarly low sensitivities to oxidation in other protein categories, to some extent, including swollenin-like proteins and proteases, both of which are implicated in early decay stages of brown rot (38). This unique overexpression and secretion of a few, oxidatively tolerant GHs involved in loosening lignocellulose (13) may be a long-overlooked adaptation that enabled brown rot fungi to succeed, evolutionarily. It will be important to extend these studies to include other brown rot fungal clades within this polyphyletic functional guild of decomposers (39). The use of refined top-down proteomics could also help gather more comprehensive data than with bottom-up proteomics with limitations in protein coverage (40). Taken alongside the broad tolerance of ROS observed in bulk enzyme extracts, however, these findings make a strong case that the secretome of the fungus *R. placenta* was tailored to endure oxidative stress inherent to the very nature of an ROS-based brown rot mechanism.

## MATERIALS AND METHODS

### Fungal cultures.

Trichoderma reesei RUT-C30 (ATCC 56765), *Trametes versicolor* A1-ATF (Forest Pathology Culture Collection, University of Minnesota, USA), and *Rhodonia placenta* MAD 698-R (ATCC 44394) were maintained on potato dextrose agar (PDA) plates for routine subculturing. For *R. placenta* and *T. versicolor*, aspen wood wafer cultures were set up as described previously (8). After 2 to 4 weeks of fungal colonization, wood wafers were harvested. Sections corresponding to early wood decay (0 to 5 mm, with 0 being the hyphal front) were sliced as previously described (12). Trichoderma reesei, which does not effectively colonize and degrade fresh wood (41, 42), was used here as a reference GH producer that is widely known for its commercial applications. For this purpose, *T. reesei* was cultured at 25°C at 150 rpm in liquid medium (2% malt extract, 0.2% yeast extract), inoculated with five edge-transferred disks (3-mm diameter) of actively growing mycelia on PDA. At day 7, the supernatant was collected by centrifugation at 4,000 × *g* at 4°C for 10 min.

### Protein extraction and sample preparation.

Colonized aspen wafer sections were extracted with 50 mM acetate buffer (0.5 M NaCl, 0.05% Tween 80 [pH 5.0]), using a ratio of buffer volume (milliliters) to sample weight (grams) of 1.5:1 (12). Extractions were performed at 80 rpm at 4°C for 24 h, and mixtures were then centrifuged at 2,500 × *g* at 4°C for 10 min. Supernatants were filtered through a 0.45-μm filter to remove any remaining suspended solids. Before enzyme analyses, extraction buffer in the crude extracts of *R. placenta* and *T. versicolor* as well as *T. reesei* supernatants were exchanged for 50 mM citrate buffer (pH 5.0) using 10-kDa-cutoff Amicon Ultra-15 centrifugal filter units (Millipore-Sigma, USA). This step allowed the removal of low-molecular-weight metabolites. The protein concentration was analyzed with a protein assay kit (Bio-Rad, USA). For proteomics, the extraction buffer included protease inhibitor Complete Ultra tablets, mini, EDTA-free, EASYpack protease inhibitor cocktail (Millipore-Sigma, USA), used according to the manufacturer’s instructions.

### Enzyme tolerance of oxidative stress.

Protein extracts obtained as described above were subjected to a range of hydroxyl radical concentrations generated via the Fenton reaction (Fe^2+^ + H_2_O_2_ → Fe^3+^ + HO^−^ + HO·) (5). Equal amounts of protein (120 μg) from each fungus were treated with FeSO_4_ at a single concentration (1 mM) and H_2_O_2_ at various concentrations (1, 5, 15, 30, and 60 mM) to match conditions in this work with those from previous work aiming to generate realistic oxidative stress during brown rot (12). Briefly, the samples were incubated with the Fenton reagents for 1 h at room temperature, and H_2_O_2_ was then inactivated by the addition of catalase (2,990 U) and further incubation for 1 h at 25°C. Three different controls were included in this experiment: control 0_A_, without Fe^2+^ or H_2_O_2_; control 0_B_, without H_2_O_2_ but with 1 mM Fe^2+^; and control 0_C_, with no H_2_O_2_ but with 1 mM Fe^2+^, with catalase added after 1 h of incubation at room temperature. We also included deglycosylation treatment of the bulk extracts (protein deglycosylation mix II, catalog number P6044S; New England BioLabs), carried out as recommended by the provider, to test whether excessive brown rot GH glycosylation (11) was responsible for ROS tolerance, as described previously by Martínek et al. (20). For proteomics, samples were treated with only FeSO_4_ (1 mM) and H_2_O_2_ (60 mM). Controls with only H_2_O_2_ were not evaluated here as we previously demonstrated that treatment with only H_2_O_2_ is not as efficient in reducing enzyme activity as the Fenton reaction is (12).

The α-l-arabinofuranosidase and α-d-galactosidase activities were measured using *p*-nitrophenyl-α-l-arabinofuranoside and *p*-nitrophenyl-α-d-galactopyranoside (Millipore-Sigma, USA), respectively (43). The released *p*-nitrophenol was quantified at 420 nm using a standard curve of *p*-nitrophenol. One enzyme activity unit was defined as the amount of enzyme needed (milligrams) to release 1 μmol of *p*-nitrophenol per min at 45°C.

### Bottom-up proteomics.

A label-free proteomics approach was carried out in this research, which allows accurate relative quantification only between samples rather than within samples. Protein samples were precipitated with a solution of chloroform-methanol (2:1) and digested with trypsin. Afterwards, the samples were separated by C_18_ reversed-phase liquid chromatography using a Waters NanoAcquity system and analyzed by mass spectrometry (MS) using a Thermo Orbitrap Fusion Lumos system. MS data were searched against the corresponding fungal databases from the Joint Genome Institute (JGI), and gene function was determined by Gene Ontology (GO) enrichment analysis with GOATOOLS (44) and manual curation. Protein quantification results were obtained with the MaxQuant software (version 1.6.1.0) with a mass error tolerance of 4.5 ppm. On the other hand, peptide quantitation and oxidative modifications were analyzed as dynamic modifications (45) with PEAKS Studio (version 10.0, build 20190129). A list of all the included PTMs is given in Table S1 in the supplemental material. Additional details are provided in Text S1.

10.1128/mBio.02040-21.1 TEXT S1 Supplemental materials and methods. Download Text S1, DOCX file, 0.02 MB. Copyright © 2021 Castaño et al. 2021 Castaño et al. https://creativecommons.org/licenses/by/4.0/ This content is distributed under the terms of the Creative Commons Attribution 4.0 International license.

10.1128/mBio.02040-21.7 TABLE S1 Oxidative modifications identified using proteomics. Download Table S1, DOCX file, 0.01 MB. Copyright © 2021 Castaño et al. 2021 Castaño et al. https://creativecommons.org/licenses/by/4.0/ This content is distributed under the terms of the Creative Commons Attribution 4.0 International license.

### Protein modeling and molecular docking.

Protein sequences of α-d-galactosidases and α-l-arabinofuranosidases were downloaded from the JGI database (https://mycocosm.jgi.doe.gov/mycocosm/home). Signal peptides in each sequence were determined using the SignalP 4.1 server (http://www.cbs.dtu.dk/services/SignalP-4.1/). When signal peptides were predicted as being present, they were cut off the sequence prior to modeling. To model proteins, we used the Modeller toolkit developed by the Max Planck Institute for Developmental Biology (https://toolkit.tuebingen.mpg.de/jobs/7727729) (46, 47). The quality of the proteins modeled in this way was validated using the online tools Verify3D, ERRAT, and Procheck, available on the SAVES server (https://servicesn.mbi.ucla.edu/SAVES/), as well as Prosa (https://prosa.services.came.sbg.ac.at/prosa.php). Proteins that did not show good scores for any of the previous metrics were iteratively refined using Refine software on the Galaxy server (http://galaxy.seoklab.org/index.html) until satisfactory values were obtained. For molecular docking, the three-dimensional (3D) structures of the *p*-nitrophenyl-α-l-arabinofuranoside and *p*-nitrophenyl-α-d-galactopyranoside substrates were downloaded from the PubChem database (https://pubchem.ncbi.nlm.nih.gov/) and subsequently energy minimized using Open Babel. Afterwards, LeDock software (http://www.lephar.com/index.htm) was used to find the conformation of the ligand in the active site. To locate the candidate active residues around which the search should be started, structural and sequence alignments were carried out using PDB reference files (accession numbers 1WD4, 2D43, 3LXA, 3S5Z, and 2XN2) from enzymes where the active sites have already been elucidated. The getbox plug-in (https://github.com/MengwuXiao/GetBox-PyMOL-Plugin) for PyMOL (http://www.pymol.org) was used to set the initial search coordinates for LeDock. Finally, protein-ligand interactions were identified using LigPlot+ (48).
