# Supplementary figures and images for: A Fungal Secretome Adapted for Stress Enabled a Radical Wood Decay Mechanism
Source: mBio. 2021 Aug 17;12(4):e02040-21. doi: 10.1128/mBio.02040-21 (PMC8406313; doi:10.1128/mBio.02040-21)

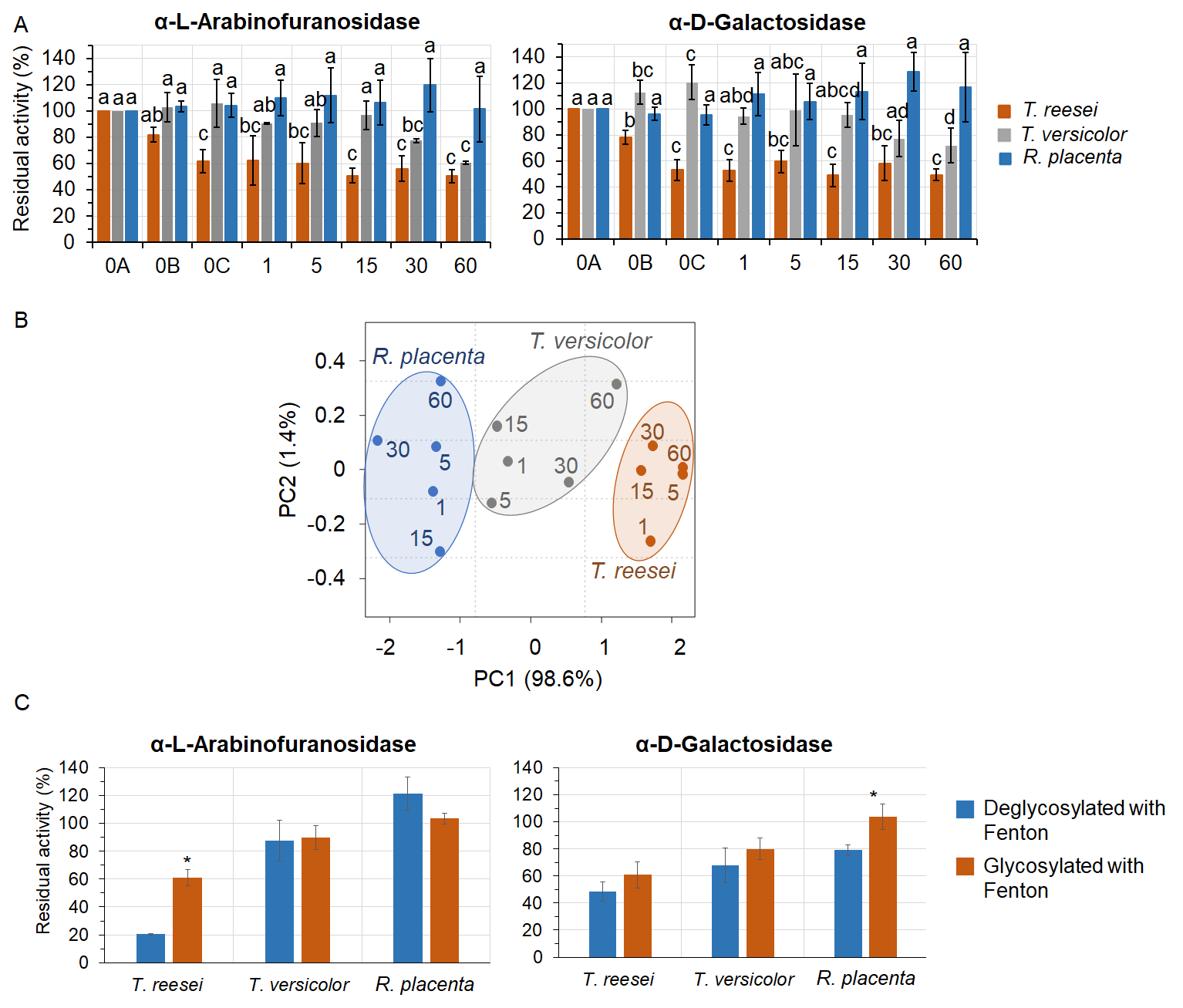

Supplement: FIG S1 [file mbio.02040-21-sf001.tif]

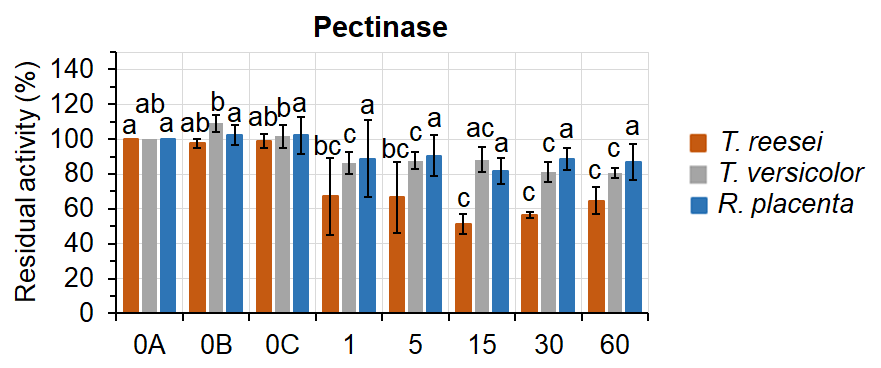

Supplement: FIG S2 [file mbio.02040-21-sf002.tif]

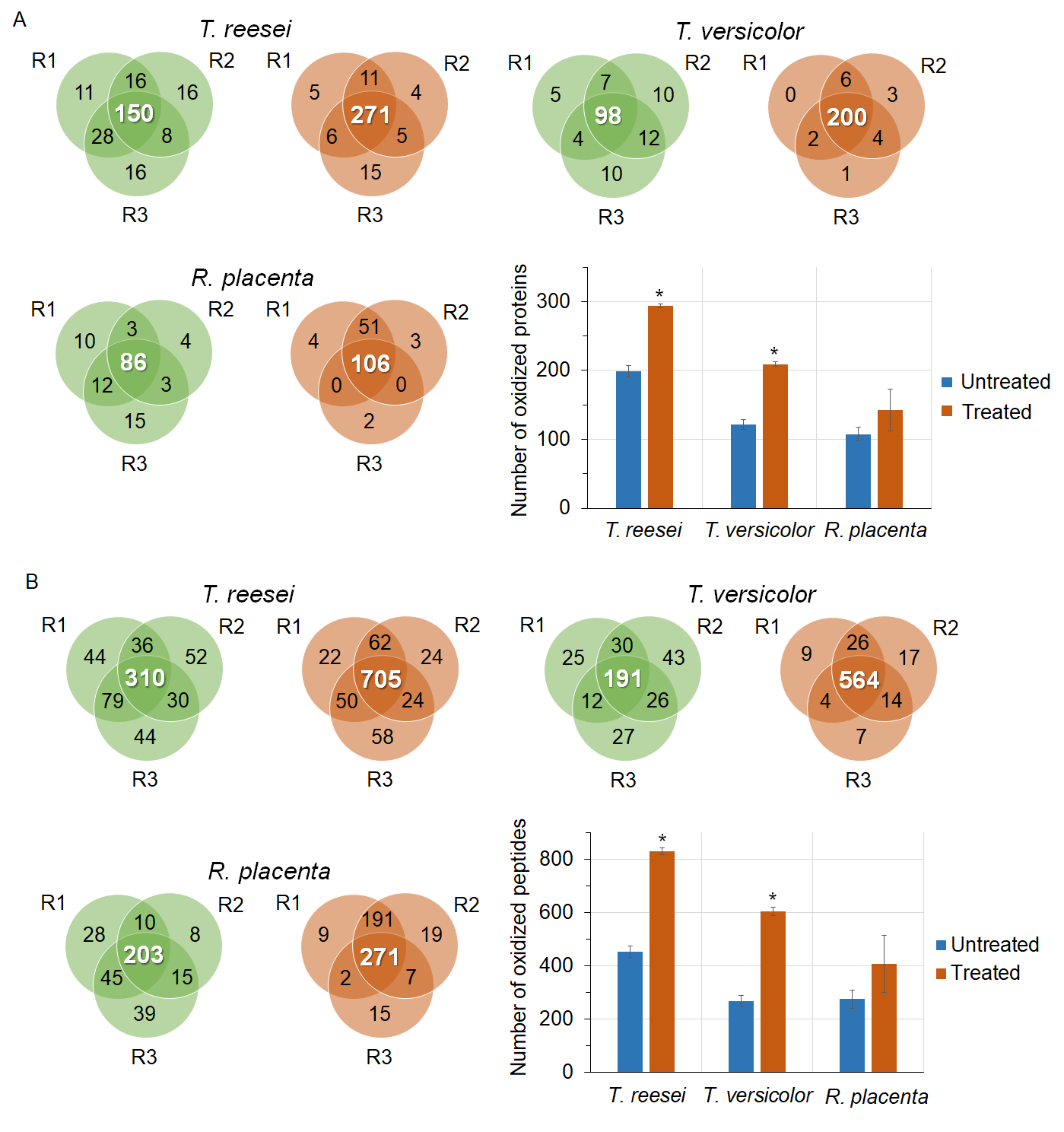

Supplement: FIG S3 [file mbio.02040-21-sf003.tif]

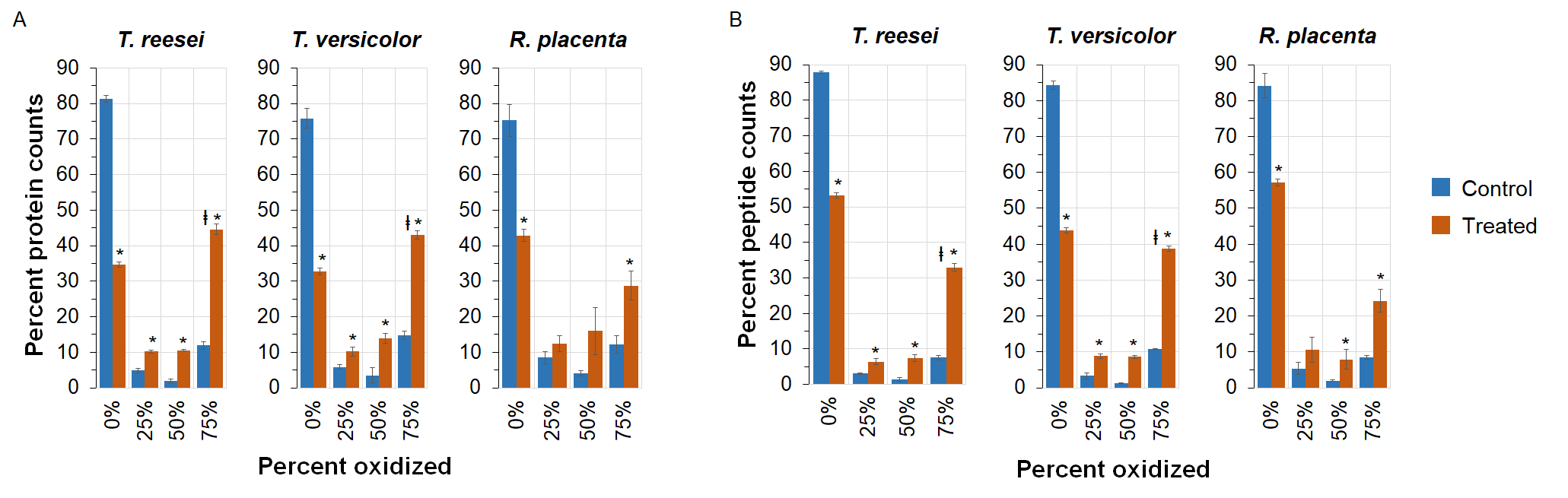

Supplement: FIG S4 [file mbio.02040-21-sf004.tif]

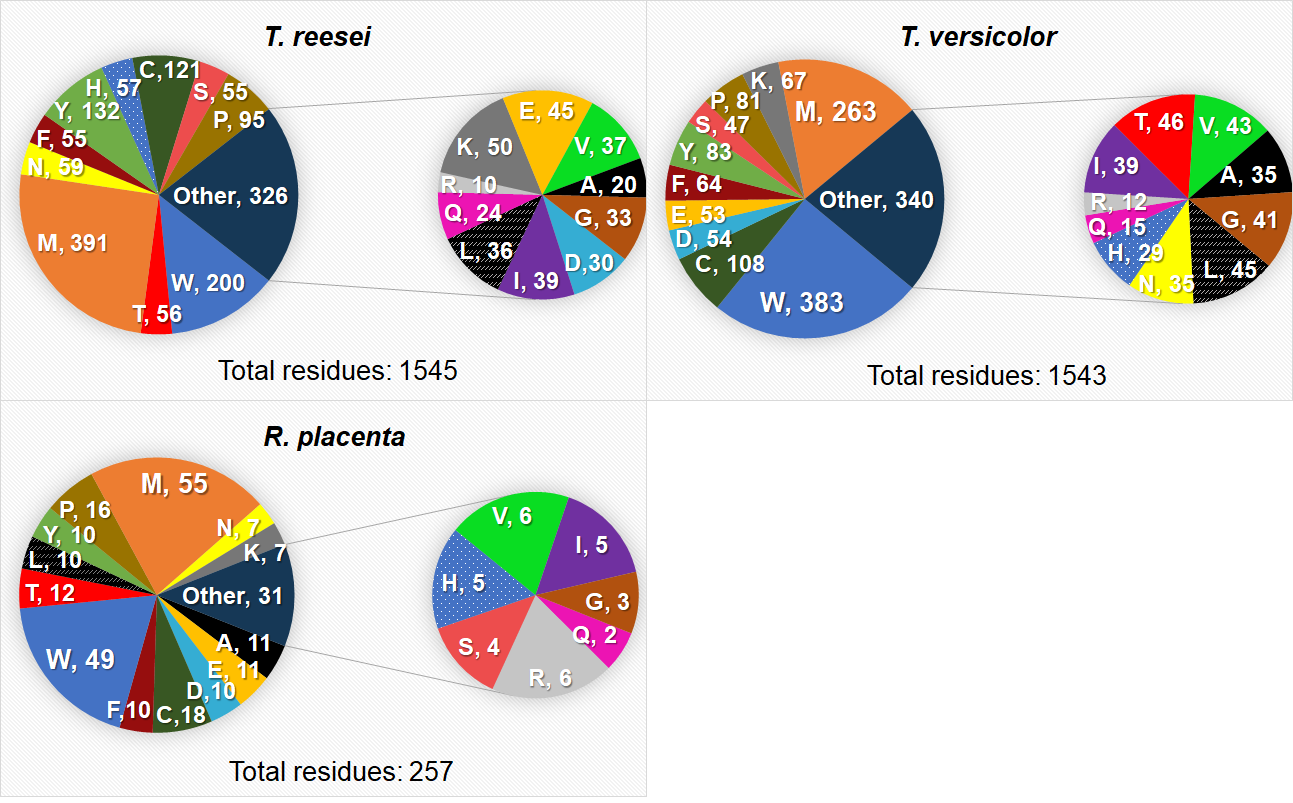

Supplement: FIG S5 [file mbio.02040-21-sf005.tif]
