# Supplementary material for: A Fungal Secretome Adapted for Stress Enabled a Radical Wood Decay Mechanism
Source: mBio. 2021 Aug 17;12(4):e02040-21. doi: 10.1128/mBio.02040-21 (PMC8406313; doi:10.1128/mBio.02040-21)
Supplement: TABLE S2 [file mbio.02040-21-st002.docx]

**Table S2**. Interacting residues in the catalytic pocket predicted through molecular docking analysis

| **Fungus** | **Enzyme** | **Binding residues** |
| --- | --- | --- |
| T. reesei | GH54-55319 | C159, C160, M178, G186, D187, G188, S189, D204, L209, E206, N207,G280, D281, S283, |
|  | GH36-124016 | W189, W326, D357, W411, R443, K476, D478, N480, D540, S520 |
|  | GH27-72632 | W19, D54, D55, Y96, C104, A105, K130, D132, C203, W205, R222, G225, D226 |
| T. versicolor | GH51-172787 | F36, E38, G104, N105, W130, E207, Y273, E328, W332, L343, Q376, I381, Q429, W430, |
|  | GH51-59914 | G231, N232, L234, A289, V290, E348, G350, N351, E352, D353, T385, H403 |
|  | GH27-60477 | W24, D59, D60, C109, Q110, D137, C179, W181, G201, D202 |
| R. placenta | GH51-100251 | N189, L191, E309, F311, Y359, I396, N399, R411, |
|  | GH27-120395 | N14, W16, D52, Y94, C102, K128, D130, D190 |
