# Supplementary material for: A Fungal Secretome Adapted for Stress Enabled a Radical Wood Decay Mechanism
Source: mBio. 2021 Aug 17;12(4):e02040-21. doi: 10.1128/mBio.02040-21 (PMC8406313; doi:10.1128/mBio.02040-21)
Supplement: TEXT S1 [file mbio.02040-21-s0001.docx]

**SI Appendix**

**Fungal enzymes adapted to enable a non-enzymatic wood decay mechanism**

Jesus Castaño^1,5^, Jiwei Zhang^1^, Mowei Zhou^2^, Chia-Feng Tsai^3^, Joon Yong Lee^2^, Carrie Nicora^3^, Jonathan Schilling^4,*^

^1^Bioproducts & Biosystems Engineering, University of Minnesota, Saint Paul, MN, USA, 55108.

^2^Environmental Molecular Sciences Laboratory, Pacific Northwest National Laboratory, Richland, WA, USA, 99352.

^3^Biological Sciences Division, Pacific Northwest National Laboratory, Richland, WA, USA, 99352.

^4^Plant & Microbial Biology, University of Minnesota, Saint Paul, MN, USA, 55108.

^5^Marine and Coastal Research Institute, Invemar, Santa Marta, Colombia, 470006.

*Corresponding author: [schillin@umn.edu](mailto:schillin@umn.edu), 1500 Gortner Avenue, Saint Paul, MN, 55108, USA

**Supporting Materials and Methods**

***Proteomics sample preparation.*** Extracted protein solutions were frozen until used for proteomics analysis following the MPLex method (1). Cold chloroform: methanol (prepared 2:1 v/v, -20°C) was mixed with sample in a 5:1 volume ratio (sample: mix) and vortexed. Samples were left on ice for 5 min, sonicated, vortexed for 5-10 seconds, and cooled on ice. Then samples were centrifuged at 12,000 x g for 5 minutes at 4 °C. The protein layers were carefully saved after removing the upper and lower layers. 1mL ice-cold MeOH was added to the protein pellet and vortexed. The suspension was centrifuged again at 12,000 x g for 5-10 minutes at 4 °C. MeOH supernatant was discarded. The tubes containing the protein pellet were inverted on a Kim wipe for a few minutes, air-dried in a sample rack for 5-10 minutes in the fume hood until the protein slightly cracks or appears completely dry. Then they were snap-frozen until digestion.

Protein pellets were mixed with 200ul of 8M urea, vortexed, pipetted harshly for ~5 min, and sonicated in a water bath until fully suspended. 10 mM dithiothreitol (DTT) was added from a 500 mM stock solution and incubated 1 h at 60 °C. 1.6mL 100mM ammonium bicarbonate was added to each tube along with 4 µg trypsin. Samples were incubated and shaken at 37 °C and 800 rpm for 3 hours. After digestion, C18 cartridge (100mg) on the automated Gilson system, and dried to ~100ul for BCA.

***Mass Spectrometry.*** 0.5 µg of digested peptides were loaded onto a liquid chromatography-mass spectrometer (LC-MS) system. The LC system is a Waters NanoAcquity equipped with in-house packed C18 reversed-phase analytical column (60 cm length, 75 μm i.d, 3 μm, 300 Å pore size, Phenomenex, Terrence, USA) and an online desalting C18 trap column (5 cm length, 100 μm i.d, same material as the analytical column). Solvent A was 0.1% formic acid in water, and solvent B was 0.1% formic acid in acetonitrile. Samples were first loaded onto the trap and flushed with 1% solvent B for 10 min. Then the trap was connected to the analytical column. The gradient started from 1% B and ramped to 35% over 100 min.

MS data were acquired on Thermo Orbitrap Fusion Lumos over 120 min for each sample. Data dependent acquisition with a cycle time of 3 sec was used. MS1 spectra were collected at 60k resolution across m/z range of 400-3000, maximum injection time of 50 ms, and AGC target 1E6. MS2 spectra with HCD were collected at 30k resolution across m/z range of 150-2000, maximum injection time of 54 ms, and AGC target of 5E5. If at least two glycan oxonium ions (204.0872, 138.0555, 168.0661, 126.055, 186.0766) were detected in the top 20 peaks in HCD (35% normalized collision energy), additional CID and EThcD spectra would be collected for the same precursor. CID (35% normalized collision energy) and EThcD (calibrated ETD time and 15% supplemental activation) spectra were collected at 50k resolution, maximum injection time 100 ms, and AGC target 5E5. Raw data are available from data depository MassIVE (https://massive.ucsd.edu) via accession MSV000085635.

***Proteomics data processing.*** MS data were searched against databases containing the corresponding fungal protein sequences (from the Joint Genome Institute) and common contaminants (trypsin and human keratins) using MaxQuant (version 1.6.1.0) and PEAKS Studio (version 10.0 build 20190129) both with false discovery rate (FDR) at 1%. Protein quantitation results were based on MaxQuant. Mass error tolerance was 4.5 ppm. Semispecific trypsin digestion was allowed. Matches between runs were enabled for label-free quantitation. Peptide quantitation and post-translation modification (PTM) profiling were based on PEAKS Studio because of the ability to specify large numbers of PTMs. Oxidative modifications (e.g. monooxidation, dioxidation, trioxidation, carbonylation) were included as dynamic modifications (2). A list with all the included PTMs is given in Supplemental table S1.

The protein and peptide abundances were normalized using Perseus. Briefly, the raw abundances were log2 transformed, and subtracted by the median. Missing values were then filled with imputation from a normal distribution. The normalized abundances were used to calculate the fold change and p values between control and treated groups. Significant changes were defined as p-value < 0.01 and log2 fold change >2.

***Gene Ontology (GO) Term Analysis for the fungi protein.*** We performed the GO enrichment analysis (GOEA) with GOATOOLS, which is a python package for GO analyses (3). We used the go-basic.obo (2019-12-09) containing 47,311 GO Terms. We employed the GOEnrichmentStudyNS function to create the GOEA objects. We created a GOEA object per each fungus by loading the gene ID and GO associations based on PANNZER annotations (PPV>= 0.4) and all the protein-coding genes of a fungus as the background gene set (4). We set up the significance cut-off of 0.05 with the Benjamini-Hochberg (BH) adjustment for the multiple test correction. The pandas and seaborn python packages were utilized for data manipulation and visualization.

**References**

1. K. E. Burnum-Jhonson, *et al.*, MPLEx: a method for simultaneous pathogen inactivation and extraction of samples for multi-omics profiling. *Analyst* **142**, 442–448 (2017).

2. I. M. Møller, A. Rogowska-Wrzesinska, R. S. . Rao, Protein carbonylation and metal-catalyzed protein oxidation in a cellular perspective. *J. Proteomics* **74**, 2228–2242 (2011).

3. D. V. Klopfenstein, *et al.*, GOATOOLS: A Python library for Gene Ontology analyses. *Sci. Rep.* **8**, 10872 (2018).

4. P. Törönen, A. Medlar, L. Holm, PANNZER2: a rapid functional annotation web server. *Nucleic Acids Res.* **46**, W84–W88 (2018).
