# Supplementary material for: A Fungal Secretome Adapted for Stress Enabled a Radical Wood Decay Mechanism
Source: mBio. 2021 Aug 17;12(4):e02040-21. doi: 10.1128/mBio.02040-21 (PMC8406313; doi:10.1128/mBio.02040-21)
Supplement: TABLE S1 [file mbio.02040-21-st001.docx]

**Table S1.** Oxidative modifications identified using proteomics

| **Mass shift** | **Oxidative Modification** |
| --- | --- |
| -2.02 | (Z)-2,3-didehydrotyrosine (Y) |
| -2.02 | 2-amino-3-oxo-butanoic_acid (T) |
| -2.02 | 3,4 didehydro lysine (K) |
| -2.02 | 3-oxoalanine (S) |
| -1.03 | Aminoadipic semialdehyde (K) |
| -17.03 | Ammonia-loss (N) |
| -32.01 | Aspartate semialdehyde (M) |
| -10.03 | Aspartylurea (H) |
| -45.02 | Carbonylation (D) |
| -59.04 | Carbonylation (E) |
| +13.98 | carbonylation unknown (E,I,K, L, Q, R, V) |
| -30.01 | Decarboxylation (D,E) |
| -33.99 | Dehydroalanine (C) |
| +63.98 | Dihydroxy-N-formylkynurenine (W) |
| 31.99 | Dioxidation (C,F,K, P, W, Y) |
| +4.98 | Formylaspargine (H) |
| +33.97 | Homocysteic acid (M) |
| +15.99 | Monooxidation (A,C,D,E, F, G, H, I, K, L, M, N, P, Q, R, S, T, V, W, Y) |
| +15.01 | Oxidation to 2-aminotyrosine (Y) |
| -43.05 | Oxidation to glutamic semialdehyde (R) |
| +19.99 | Oxidation to hydroxykynurenin (W) |
| +3.99 | Oxidation to kynurenin (W) |
| +13.98 | Oxidation to oxolactone (W) |
| +13.98 | Oxidation to pyroglutamic acid (P) |
| -30.01 | Oxidation to pyrrolidinone (P) |
| -17.99 | Oxoalanine (C) |
| -27.99 | Pyrrolidone (P) |
| +29.97 | Quinone (W,Y) |
| +47.98 | TriOxidation (C,F,W) |
| +28.99 | S-nitrosylation (C) |
